# A case of malignant mesothelioma in a young patient with childhood leukaemia who had received total body irradiation

**DOI:** 10.1093/omcr/omac151

**Published:** 2023-05-30

**Authors:** Redwan Hayat, Saad Ahmed, Rekha Badiger, Shi Han Lee, Adam Peryt, David Gilligan, Zubair Khalid

**Affiliations:** General Practice, Broomfield Hospital, Chelmsford, UK; Acute Medicine, Colchester General Hospital, Colchester, UK; Respiratory Medicine, Colchester General Hospital, Colchester, UK; Respiratory Medicine, Colchester General Hospital, Colchester, UK; Cardiothoracic Surgery, Royal Papworth Hospital, Cambridge, UK; Clinical Oncology, Cambridge University Hospitals, Cambridge, UK; Respiratory Medicine, Broomfield Hospital, Chelmsford, UK

## Abstract

This case report explores a 34-year-old male diagnosed with mesothelioma who had no known risk factors. The patient initially was treated for empyema with antibiotics but later represented to hospital with worsening symptoms. He underwent a surgical Video-assisted thoracoscopic surgery procedure and lung biopsy, which revealed a diagnosis of mesothelioma. The young age of the patient as well as absence of significant risk factors for mesothelioma made the diagnosis unexpected. The patient had total body irradiation (TBI) therapy for leukaemia as a child, which increases the risk of developing cancer. However, there are limited studies exploring the risk of pleural mesothelioma post-TBI. Young patients who represent to hospital, with limited response to initial treatment, and suspicious radiological features should be considered for lung biopsy to reduce the risk of a missed diagnosis. Patients with a background of TBI should also be considered for follow-up to monitor for any subsequent malignancy.

## INTRODUCTION

Mesothelioma is a malignant tumour of the mesothelium, most commonly presenting in the pleura. In the UK, it accounted for 1% of all deaths between 2016 and 2018. Mesothelioma is strongly associated with asbestos fibre exposure, and patients often have a background of occupational exposure. Other non-asbestos mineral fibres and therapeutic radiation are less common risk factors for developing mesothelioma [1]. Almost 20% of patients diagnosed with mesothelioma have no prior history of asbestos exposure. There are other identified causes of mesothelioma including: erionite mineral fibre, carbon nanotubes, irradiation treatment, thorium dioxide, viruses including the avian leukosis virus and also chronic serosal inflammation, such as that seen in familial mediterranean fever and chronic empyema [2]. The presentation of symptoms can be as long as 40 years from asbestos exposure and deaths in the UK from mesothelioma commonly occurred between the ages of 85 and 89 [3].

This case report discusses an atypical presentation of pleural mesothelioma in a young patient in his 30s with no significant risk factors and absence of asbestos tissue in the body. The report considers total body irradiation (TBI) therapy contributing towards the diagnosis of mesothelioma and the clinical manifestations in this young patient.

## CASE PRESENTATION

**Figure 1:**
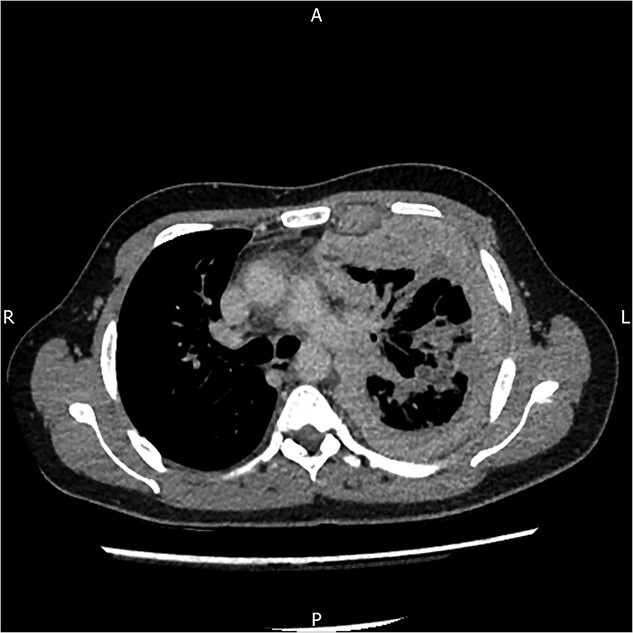
CT scan of the thorax that shows left sided pleural thickening. CT scan of the thorax.

A 34-year-old male patient presented to hospital with a short history of persistent cough, shortness of breath and fever. The patient has a background of leukaemia as a child for which treatment included TBI. He also had thyroidectomy for thyroid cancer and a history of mental health issues. There is no family history of note. The patient reported having symptoms for three weeks prior to admission but no history of chest pain. On examination, he was noted to have reduced air entry on the left and was not clubbed. He was a non-smoker and there was no history of asbestos exposure. Blood results did not show raised inflammatory markers. The patient had a CT scan of the thorax which revealed marked pleural thickening with a left sided effusion (Figure 1). The patient refused pleural aspiration during this admission. The working diagnosis was empyema for which he was treated with intravenous antibiotics. The patient refused surgical intervention during this admission and his background of anxiety may have contributed to this decision. He was discharged with a prolonged course of oral antibiotics, with a view to early follow up.

Two months after his initial presentation to hospital, he was re-admitted due to worsening shortening of breath and chest pain. A repeat CT scan showed significant progression of his left sided pleural thickening with a new right sided effusion. Blood results again did not demonstrate a significant rise in infection markers. Microbiological and cytological analysis of pleural fluid did not reveal an obvious diagnosis (Table 1).

**Table 1:** Results of aspiration of the left pleural fluid

Pleural/serum protein	37/60 g/L
LDH	>1200 U/L
pH	7.269
Glucose	1.7 mmol/L
Microbiology and cytology	No organisms seen, no growth

Results of aspiration of the left pleural fluid.

**Figure 2:**
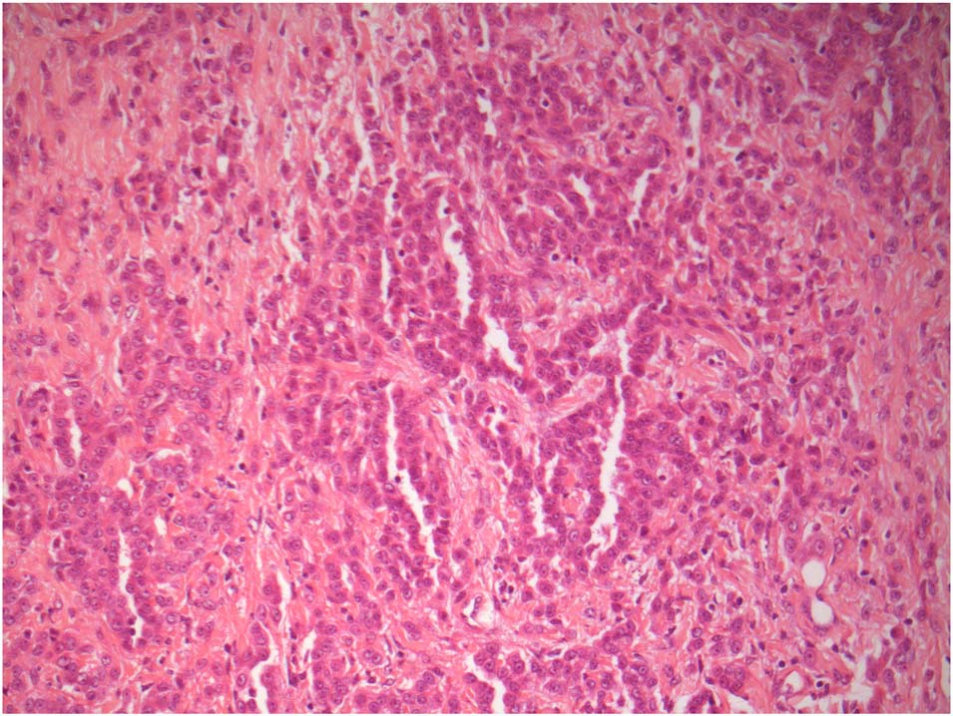
H&E x100—Parietal pleural tissue extensively infiltrated by epithelioid tumour cells forming cords, nests and tubular structures. The tumour cells possess mild to moderately pleomorphic nuclei, visible nucleoli and eosinophilic cytoplasm. H&E stain microscopy image of the left lung parietal pleura tissue

**Figure 3:**
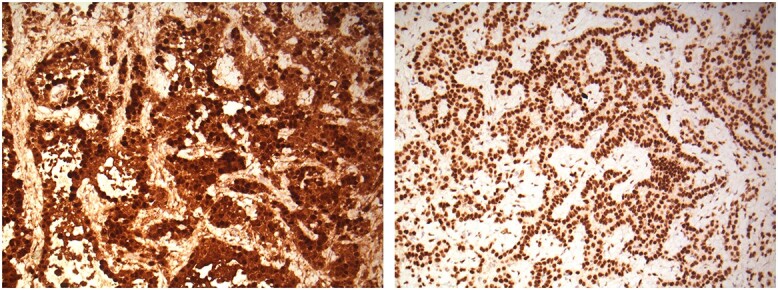
Calretinin & WT1 x200—Parietal pleural sample. The tumour is diffusely positive for the mesothelial markers calretinin (nuclear and cytoplasmic positivity) and WT1 (nuclear staining). Calretinin and WT1 stain microscopy images of the left lung parietal pleura tissue.

With further counselling, he underwent a left Video-assisted thoracoscopic surgery procedure with pleural biopsy. The appearances were noted to be atypical for empyema as well as malignancy. There were post-inflammatory changes seen with a significantly thickened pleura, but no frank pus visible. Biopsies were taken from left lung parietal pleura, which confirmed an unexpected diagnosis of epithelioid mesothelioma T4 N1 M0 (Figures 2 & 3).

Unfortunately, the patient was deemed unsuitable for palliative chemotherapy as his performance status had now deteriorated to 3, and he was managed with supportive care. Sadly, the patient passed away 17 months after his initial presentation at the age of 35.

## DISCUSSION

The young age of the patient along with the absence of significant asbestos-related risk factors for mesothelioma made the diagnosis of mesothelioma unexpected. Other factors should be explored which may have contributed to the development of mesothelioma.

The incidence of mesothelioma in those aged under 35 years old in the UK was 4 per 100 000, with the trend of incidence significantly increasing with age [4]. The young presentation of the patient without any known asbestos exposure would have suggested a low clinical suspicion of mesothelioma.

The patient underwent TBI therapy for the treatment of leukaemia during his childhood. The risk of developing cancer is increased after TBI. The latency of mesothelioma development after TBI was 29 years in our patient. The level of radiation along with age at which treatment was received can influence the clinical risk. A study looking at patients over a 45-year period found that higher dose radiation as well as treatment under the age of 20 significantly increased the risk of developing cancer [5]. A further study also demonstrates that pulmonary toxicity increases after TBI [6].

There is a difference in risk of secondary carcinogenesis between local radiation therapy and total body irradiation. A study looking at leukaemia in patients with cervical cancer who received local radiotherapy found that the risk of leukaemia increased two folds (RR = 2.0; 90% confidence interval = 1.0-4.2) [7]. The risk of malignant neoplasms increased by 2.8-fold after TBI (RR = 2.8; 95% confidence interval = 2.6-3.1) [5]. The study suggested lifelong monitoring for early detection of secondary carcinogenesis following TBI. There have been reported cases of peritoneal mesothelioma after TBI, however studies looking at reported cases of pleural mesothelioma are limited [8, 9]. The British childhood cancer survivor study that looked at outcomes of children diagnosed with cancer who may have received cancer treatment did not report any cases of pleural mesothelioma after TBI [10]. A systematic literature search has not shown any similar prior cases and, to the best of our knowledge, our case is the first of its kind.

The young age of onset of mesothelioma in this patient is an uncommon finding and the diagnosis could be missed or delayed in the clinical setting. In young patients diagnosed with clinical features of extensive pleural thickening, with a low suspicion of an infective aetiology, radiological investigation and subsequent biopsy should be considered early. Lifelong monitoring post-TBI therapy is also important for the early detection of secondary carcinogenesis. Although the risk of cancer is increased in patients with a background of TBI, further research into the risk of developing pleural malignant mesothelioma after TBI would be useful to guide clinical management.

## Data Availability

The authors confirm that the data supporting the findings of this study are available within the article.
